# Two novel conserved linear B-cell epitopes identified in the S2 subunit of the infectious bronchitis virus spike protein

**DOI:** 10.1016/j.virusres.2025.199657

**Published:** 2025-11-03

**Authors:** Liwei Zhang, Yingfei Li, Xuehui Zhang, Jing Zhao, Guozhong Zhang, Ye Zhao

**Affiliations:** a National Key Laboratory of Veterinary Public Health Security, College of Veterinary Medicine, China Agricultural University, Beijing 100193, China; b Key Laboratory of Animal Epidemiology of the Ministry of Agriculture, College of Veterinary Medicine, China Agricultural University, Beijing 100193, China

**Keywords:** Infectious bronchitis virus, Spike protein, S2 subunit, Monoclonal antibody, B-cell epitope

## Abstract

Infectious bronchitis (IB), caused by the infectious bronchitis virus (IBV), is a contagious respiratory disease of chickens that poses a serious threat to the poultry industry worldwide. In this study, four monoclonal antibodies (mAbs) targeting the heptad repeat 2 (HR2) region of the spike protein S2 subunit were generated through mouse immunization, hybridoma cell fusion, and clonal purification. Western blot and indirect immunofluorescence assays confirmed that all four mAbs specifically recognized IBV. Epitope identification revealed two novel linear B-cell epitopes: ^1040^KWWND^1044^, recognized by mAbs 6A6 and 6E2; and ^1046^KHELPDF^1052^, recognized by mAbs 6A1 and 6A9, which are conserved among different IBV lineages. Furthermore, both epitopes are exposed on the surface of the spike protein, suggesting their potential as immunologically relevant targets. This study contributes to further elucidating the structure and function of the IBV S2 subunit and provide assistance for the development of IBV diagnostic technology.

## Highlights

• Four monoclonal antibodies against S2 subunit of IBV spike protein were prepared.
• Two novel epitopes of ^1040^KWWND^1044^ and ^1046^KHELPDF^1052^ were identified.
• The epitope ^1046^KHELPDF^1052^ is highly conserved among different IBV genotypes.
• Both epitopes are located within the heptad repeat 2 region and are surface-exposed.

## 1 Introduction

Infectious bronchitis (IB) is an acute, highly contagious respiratory disease in chickens caused by infectious bronchitis virus (IBV) (Legnardi et al., 2020). IBV was first reported in North Dakota, USA, and has since spread worldwide (Schalk and Hawn, 1931). The virus exhibits significant genetic diversity, encompassing multiple genotypes and lineages (Zhao et al., 2023). Among these, GⅠ−1 (Mass-type), GⅠ−13 (793/B-type) and GⅠ−19 (QX-type) lineages are prevalent in many countries, with the QX-type strain being the most widespread (Guo et al., 2025; Kim et al., 2025). Due to continuous evolution through mutation and recombination, IBV generates new serotype strains, which complicates clinical prevention and control efforts and causes significant economic losses to the global poultry industry (Rohaim et al., 2020).

IBV is a member of the genus Gammacoronavirus within the family Coronaviridae, order Nidovirales (Hassan et al., 2019). It is an enveloped, positive-sense, single-stranded RNA virus with a 27.6 kb genome containing 10 open reading frames (ORFs). The virus encodes four structural proteins: spike protein (S), envelope protein (E), membrane protein (M), and nucleocapsid protein (N), along with several non-structural proteins (Lin and Chen, 2017). The spike protein forms trimers on the viral surface and acts as the main antigen recognized by the host immune system during IBV infection or vaccination (Wickramasinghe et al., 2014). It plays a key role in triggering B-cell and T-cell responses and stimulating protective immunity in the host (Bande et al., 2016). During biosynthesis, the spike protein of IBV is cleaved into S1 and S2 subunits by furin at a conserved polybasic cleavage site (Winter et al., 2008; Yamada and Liu, 2009). The S1 subunit harbors the receptor-binding site, which mediates viral attachment and receptor recognition on the cell surface, whereas the S2 subunit facilitates fusion between the viral and host cell membranes (Winter et al., 2008; You et al., 2023). Due to sustained immune pressure and RNA genome replication characteristics, the S1 gene evolves rapidly, leading to poor cross-protection by neutralizing antibodies between different genotypes (Zhao et al., 2016). In contrast, the S2 gene is more conserved and provides an important target for developing broad-spectrum antibodies.

Only a few antigenic epitopes have been reported within the S2 subunit of the IBV spike protein. Epitopes in the fusion peptide (686–697 aa and 692–703 aa) have been identified, but they do not induce neutralizing antibodies against IBV(Andoh et al., 2018). A region at the N-terminus of the S2 subunit (566–584 aa) contains a linear neutralizing epitope that can induce an effective antibody response following immunization, although the exact sequence remains undefined (Andoh et al., 2018). Therefore, further identification of conserved epitopes in the S2 subunit would provide valuable information for future studies.

In this study, we generated four mAbs against the S2 subunit of IBV spike protein and identified two novel conserved linear epitopes recognized by these antibodies. Our findings provide important targets for studying spike protein function and developing serological diagnostic assays, thereby contributing to improved detection of diverse IBV strains.

## 2 Materials and methods

### 2.1 Animals and ethics statement

Female BALB/c mice (6–8 weeks old) were purchased from SPF Biotechnology Co., Ltd. (Beijing, China), and SPF embryonated chicken eggs (ECEs) were obtained from Beijing Boehringer Ingelheim Vital Biotechnology Co., Ltd. (Beijing, China). Animal experiments were approved by the Animal Welfare and Ethics Review Committee of China Agricultural University (Approval No AW11704308).

### 2.2 Viruses and cells

QX-like IBV: SD strain (GenBank KY421673) and YN strain (GenBank JF893452.2), TW-like IBV: GD strain (GenBank DQ646405), and Mass-like IBV: M41 strain (GenBank: DQ834384) were stored in our laboratory and grown in 10-day-old SPF ECEs. Primary chicken embryo kidney (CEK) cells were isolated from 18-day-old SPF ECEs. CEK, Vero E6, and SP2/0 cells were grown in DMEM containing 10 % FBS and 1 % penicillin-streptomycin (Gibco, USA). All cells were grown at 37 °C in a 5 % CO₂ incubator.

### 2.3 Prokaryotic expression and purification of S_860–1088_

Amino acids 860–1088 of the spike protein, which showed high homology, was selected as the target antigen. To generate the recombinant expression construct, this sequence was amplified by PCR from the full-length cDNA of IBV YN strain and subsequently cloned into the pET-28a prokaryotic expression vector using seamless cloning methodology (Uniclone One Step Seamless Cloning Kit, Genesand Biotech, Beijing, China), yielding the recombinant vector pET28a-S_860–1088_. After confirmation by Sanger sequencing, the vector was transformed into *Escherichia coli* BL21 (DE3). Recombinant protein expression was induced by treatment with 0.5 mM isopropyl β-d-1-thiogalactopyranoside (IPTG) at 16 °C for 12 h. The inclusion bodies obtained after ultrasonic disruption of the bacterial suspension were solubilized in 8 M urea. The recombinant protein was then purified by His-tag affinity chromatography and subsequently verified by Western blotting.

### 2.4 Immunization of animals

Three female BALB/c mice (6–8 weeks old) were immunized subcutaneously at multiple sites with 60 μg of purified S_860–1088_ recombinant protein emulsified 1:1 (v/v) with Freund's complete adjuvant (Sigma-Aldrich, USA). Mice received booster immunizations every two weeks using 60 μg of purified S_860–1088_ recombinant protein emulsified 1:1 (v/v) with Freund's incomplete adjuvant (Sigma-Aldrich, USA). Four days after the third immunization, blood was collected by retro-orbital bleeding and serum was separated. The mouse with the highest antibody titer received a final boost of 100 μg purified S_860–1088_ recombinant protein by intraperitoneal injection.

### 2.5 Monoclonal antibody preparation

Mice were sacrificed 7 days after the booster immunization, and splenocytes were collected for fusion with SP2/0 cells at a 10:1 ratio using PEG4000 (Sigma-Aldrich, USA). Fused cells were centrifuged at 800 × *g* for 5 min, resuspended in hypoxanthine-aminopterin-thymidine (HAT) selective medium, and seeded into 96-well plates containing mouse peritoneal macrophages as feeder cells, which had been prepared one day before fusion. The plates were then incubated at 37 °C with 5 % CO_2_ for 10 days, after which hybridoma supernatants were screened by IFA and positive clones were subcloned three times through limiting dilution. To generate ascitic fluid, nine-week-old female BALB/c mice were primed by intraperitoneal injection of 400 μL Freund's incomplete adjuvant 7 days before hybridoma cell inoculation. Each mouse was intraperitoneally injected with 10^6^ hybridoma cells, and ascitic fluid was collected using sterile needles one week later and stored at −40 °C. The specificity of the obtained mAbs was further confirmed by WB and IFA.

### 2.6 Indirect immunofluorescence assay (IFA)

CEK cells from 18-day-old ECEs were seeded in 24-well plates one day before infection. Once cells reached 80 % confluence, they were infected with virus, and at 36 h post-infection (hpi), when cytopathic effects (CPE) became evident, the medium was removed, and cells were washed three times with PBS. Cells were fixed with 200 μL acetone:ethanol (3:2) fixative at −20 °C for 15 min, followed by three washes with PBST. Immune sera or mAbs (diluted 1:4000) and/or anti-IBV nucleocapsid (N) polyclonal antibody (laboratory-made, diluted 1:1000) were applied in 5 % skim milk and incubated overnight at 4 °C. After three PBST washes, Alexa Fluor 488-conjugated anti-mouse IgG (*H* + *L*) (diluted 1:1000) and/or Alexa Fluor 555-conjugated anti-rabbit IgG (*H* + *L*) (Cell Signaling Technology, USA, diluted 1:1000) were added as secondary antibodies and incubated at 37 °C for 1 h. After three more PBST washes, cell nuclei were stained with DAPI (Sigma-Aldrich, USA) for 10 min at room temperature and washed three times with PBST. Fluorescence images were captured using a Nikon inverted fluorescence microscope.

### 2.7 Western blotting (WB)

CEK cells were infected with IBV at an MOI of 0.1. At 24 hpi, cells were washed three times with pre-chilled PBS and lysed in radio-immunoprecipitation assay (RIPA) buffer containing protease inhibitors on ice for 15 min. Lysates were centrifuged at 13,300 × *g* for 10 min, and supernatants were mixed with loading buffer and boiled for 10 min. Proteins were separated by SDS-PAGE and either stained with Coomassie Brilliant Blue or transferred to PVDF membranes. PVDF membranes were blocked with 5 % skim milk for 2 h at room temperature, washed three times with TBST, then incubated overnight at 4 °C with primary antibodies: mAbs (diluted 1:4000 in 5 % skim milk) and anti-IBV nucleocapsid (N) polyclonal antibody (laboratory-made, 1:1000). After three TBST washes, membranes were incubated with HRP-conjugated goat anti-mouse IgG and HRP-conjugated goat anti-rabbit IgG secondary antibodies (diluted 1:10,000 in PBS, Bioss, China) for 1 h at room temperature. Finally, ECL substrate (Beyotime, Shanghai, China) was added and membranes were imaged using a ChemiDoc MP system (Bio-Rad, California, USA).

### 2.8 Antigenic epitope identification

Overlapping fragments spanning amino acids 860–1088 of the spike protein were generated by site-directed mutagenesis and cloned into the pCMV-FLAG-GST vector. Key primers are listed in Table 1. Constructed plasmids were transfected into VERO cells, and epitopes recognized by mAbs 6A6, 6E2, 6A1, and 6A9 were identified by WB and IFA.

**Table 1:** Key primers for spike protein fragment cloning.

Segment	Primers	Sequences (5′−3′)	Positions (Amino Acid)
S1	S1-F	CAAAAGGAGGCGGGGGTAGTGCGCAACTTGATGCAATTCAAGC	860–1088
	S1-R	CGCGGCCGCGGTACCTCGAGTTACAGTTCTTCAAGGTCTATAATGGAGTC	
S2	S2-F	CAAAAGGAGGCGGGGGTAGTGCGCAACTTGATGCAATTCAAGC	860–970
	S2-R	CGCGGCCGCGGTACCTCGAGTTATGCATACTGACTAGCATTAGCAGGC	
S3	S3-F	CAAAAGGAGGCGGGGGTAGTTTTGTTAATGTTACTGCAATAGTGGG	950–1045
	S3-R	CGCGGCCGCGGTACCTCGAGTTAAGTGTCATTCCACCATTTCGACAA	
S4	S4-F	CAAAAGGAGGCGGGGGTAGTACATTTGTAGAAGATGACGATTTTGATTT	1025–1088
	S4-R	CGCGGCCGCGGTACCTCGAGTTACAGTTCTTCAAGGTCTATAATGGAGTC	
S5	S5-F	CAAAAGGAGGCGGGGGTAGTACATTTGTAGAAGATGACGATTTTGAT	1025–1045
	S5-R	CTCGGTCGACCGAATTCTTAAGTGTCATTCCACCATTTCGACA	
S6	S6-F	GCGGGGGTTGGAATGACACTTAAGAATTCGGTCGAC	1042–1045
	S6-R	AGTGTCATTCCAACCCCCGCCTCCTTTTGGAGG	
S7	S7-F	GACTAAGAATTCGGTCGACCGAGATCTCTC	1040–1044
	S7-R	GGTCGACCGAATTCTTAGTCATTCCACCATTTACCCCC	
S8	S8-F	AATTAAGAATTCGGTCGACCGAGATCTCTC	1040–1043
	S8-R	GGTCGACCGAATTCTTAATTCCACCATTTACCCCCGC	
S9	S9-F	CAAAAGGAGGCGGGGGTAGTACATTTGTAGAAGATGACGATTTTGATTT	1025–1060
	S9-R	CGCGGCCGCGGTACCTCGAGTTAAGGTACTGTGTAATTGAAGTCGTC	
S10	S10-F	GCTACCAGATTAACTCGAGGTACCGCGGCC	1046–1051
	S10-R	CCTCGAGTTAATCTGGTAGCTCATGCTTACTACCC	
S11	S11-F	CCAGATTTTTAACTCGAGGTACCGCGGCC	1046–1052
	S11-R	ACCTCGAGTTAAAAATCTGGTAGCTCATGCTTACTACCC	

F: forward primer; R: reverse primer.

### 2.9 Biological information analysis

Amino acid conservation within residues 860–1088 of the spike protein was analyzed using MegAlign software (version 7.1.0, DNAstar Inc, Madison, WI, USA). Structural features were assessed using DNAStar Protean software (version 7.1.0, DNAstar Inc, Madison, WI, USA). Amino acid sequences of 800 IBV strains were downloaded from NCBI database and epitope conservation was assessed using Geneious Prime software (Version 2023.2.1, Biomatters Ltd., Auckland, New Zealand). Sequence conservation was visualized using WebLogo (https://weblogo.threeplusone.com/create.cgi). IBV spike protein structure was predicted using AlphaFold 3 (https://alphafoldserver.com/), and epitopes positions were mapped onto the predicted structure using PyMol software (Version 3.1, Schrödinger, LLC, New York, NY, USA) to analyze spatial features.

## 3 Results

### 3.1 Expression and purification of recombinant proteins

**Fig. 1:**
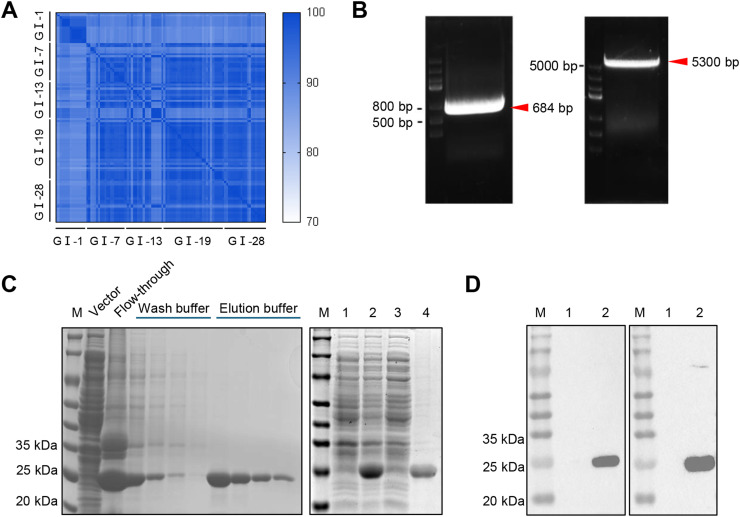
Expression and purification of the S_860–1088_ protein. (A) Sequence similarity matrix of the S2 subunit region (860–1088 aa) from five major IBV lineages (GI-1, GI-7, GI-13, GI-19 and GI-28) isolated in China. The color scale indicated similarity percentages. (B) Agarose gel electrophoresis of PCR products. Lane 1: amplified DNA fragment encoding S_860–1088_ amino acids. Lane 2: pET-28a vector digested with BamH I and Xho I. (C) SDS-PAGE analysis of recombinant S_860–1088_ protein expression. Left panel: protein purification steps. Lane M: protein marker; Vector: empty vector control; Flow-through: unbound proteins; Wash buffer: wash fraction; Elution buffer: purified target protein. Right panel: SDS-PAGE analysis of protein expression in bacterial lysates. Lane M: protein marker; Lane 1: empty vector control; Lane 2: pellet after sonication; Lane 3: supernatant after sonication; Lane 4: purified recombinant S_860–1088_ protein. (D) WB confirmation of recombinant S_860–1088_ protein identity. The left panel was probed with anti-His antibody, and the right panel with anti-YN antiserum. Lane M: protein marker. Lane 1: empty vector control; Lane 2: purified recombinant S_860–1088_ protein.

To evaluate the conservation of amino acids 860–1088 in the IBV spike protein, we compared strains from major IBV lineages isolated in China. Amino acid sequence alignment showed that this region was highly conserved across different lineages (Fig. 1A). Based on the full-length cDNA of the YN strain, strain-specific primers were designed, and the DNA fragment encoding S_860–1088_ was amplified by PCR and cloned into the pET-28a expression vector (Fig. 1B). SDS-PAGE analysis confirmed that the recombinant S_860–1088_ protein was successfully expressed in *E. coli*. The recombinant S_860–1088_ protein had an apparent molecular weight of about 25 kDa. Most of the expressed protein was found in the pellet after sonication, and the purified recombinant S_860–1088_ protein showed high purity (Fig. 1C). WB analysis further confirmed the identity of the recombinant protein, showing that it was specifically recognized by both anti-His antibody and anti-YN antiserum (Fig. 1D). These results demonstrate the conservation of S_860–1088_ and the successful preparation of its recombinant protein.

### 3.2 Production of mAbs against IBV spike protein S2 subunit

**Fig. 2:**
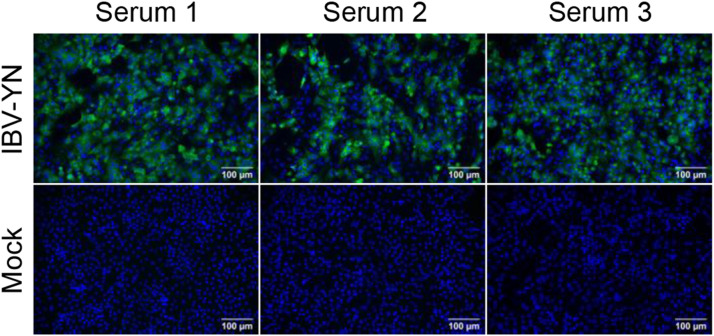
Production of mAbs. (A) IFA showing reactivity of sera from three immunized mice against CEK cells with or without IBV infection. Mouse sera served as primary antibodies, and Alexa Fluor 488-conjugated anti-mouse IgG (*H* + *L*) as secondary antibody. Scale bar: 100 μm.

Sera from three immunized mice were screened by IFA against IBV-infected CEK cells (Fig. 2). Splenocytes from the mouse with the strongest signal were fused with SP2/0 cells, after which the resulting hybridoma cells were subjected to three rounds of limiting-dilution subcloning. These results indicate that S_860–1088_ possesses good immunogenicity and stable hybridoma cell lines producing antibodies specific to the IBV spike protein S2 subunit were obtained (6A6, 6E2, 6A1, and 6A9).

### 3.3 Identification of epitopes recognized by mAbs

**Fig. 3:**
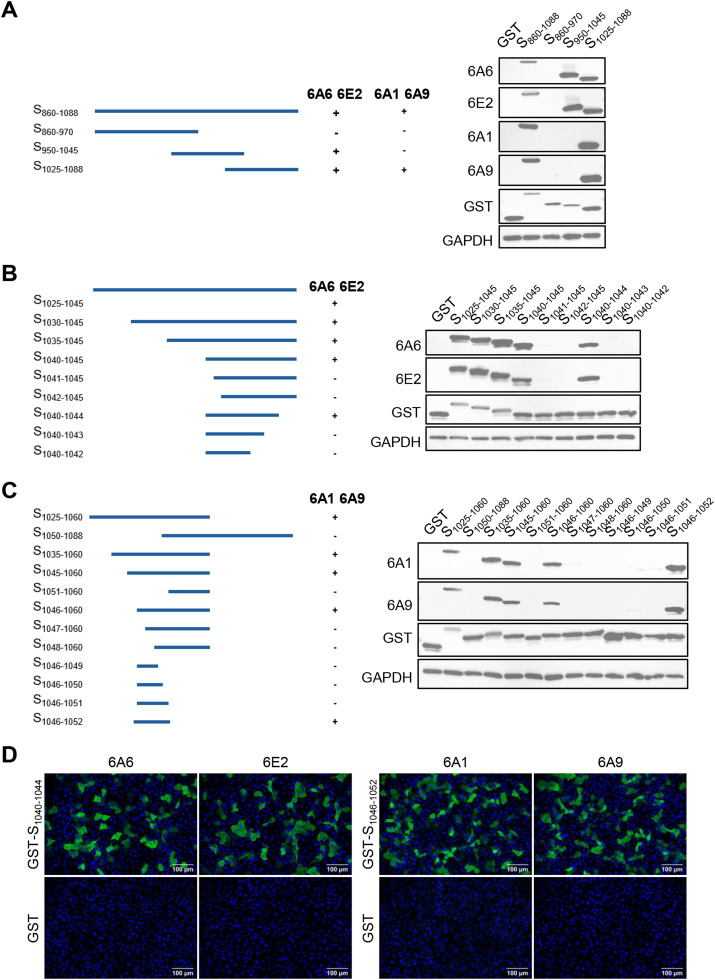
Identification of linear epitopes recognized by mAbs using WB and IFA. (A-C) Truncation scheme for S_860–1088_ (left) and corresponding WB showing recognition of GST-tagged fragments expressed in Vero E6 cells (right). GAPDH was used as an internal control. "+" indicates positive recognition; "-" indicates no recognition. Primary antibodies: mAbs 6A6, 6E2, 6A1and 6A9; secondary antibody: HRP-conjugated goat anti-mouse IgG. (D) IFA assessment of mAbs recognition of minimal epitopes. Epitopes ^1040^KWWND^1044^ and ^1046^KHELPDF^1052^ were cloned into pCMV-FLAG-GST vector and transfected into VERO cells. GST alone served as negative control. Primary antibodies: prepared mAbs; secondary antibody: Alexa Fluor 488-conjugated anti-mouse IgG (*H* + *L*).

To identify the epitopes recognized by mAbs 6A6, 6E2, 6A1, and 6A9, a series of GST-tagged truncated spike proteins were expressed in Vero E6 cells and their recognition by each antibody was examined using WB. The results showed that mAbs 6A6 and 6E2 recognized S_860–1088_, S_950–1045_, and S_1025–1088_ fragments, while mAbs 6A1 and 6A9 recognized only S_860–1088_ and S_1025–1088_ fragments but not the S_950–1045_ fragment (Fig. 3A). These results suggest that the epitopes for mAbs 6A6 and 6E2 are located within amino acid residues 1025–1045 of the spike protein, while those for mAbs 6A1 and 6A9 are located within residues 1025–1088. To precisely identify the minimal amino acid sequences of these linear B-cell epitopes, a stepwise truncation approach was used. The findings revealed that mAbs 6A6 and 6E2 recognized the S_1040–1044_ fragment but not S_1041–1045_ or S_1040–1043_, indicating the minimal epitope is ^1040^KWWND^1044^ (Fig. 3B). Similarly, mAbs 6A1 and 6A9 recognized the S_1046–1052_ fragment but not S_1046–1051_ or S_1047–1060_, defining the minimal epitope as ^1046^KHELPDF^1052^ (Fig. 3C). Both minimal epitopes were cloned into a GST expression vector, and IFA showed that all 4 mAbs bound to their corresponding epitopes (Fig. 3D). These results demonstrate that the four generated monoclonal antibodies targeting the IBV S2 subunit recognize two distinct linear B-cell epitopes, ^1040^KWWND^1044^ and ^1046^KHELPDF^1052^.

### 3.4 Bioinformatics analysis of identified epitopes

**Fig. 4:**
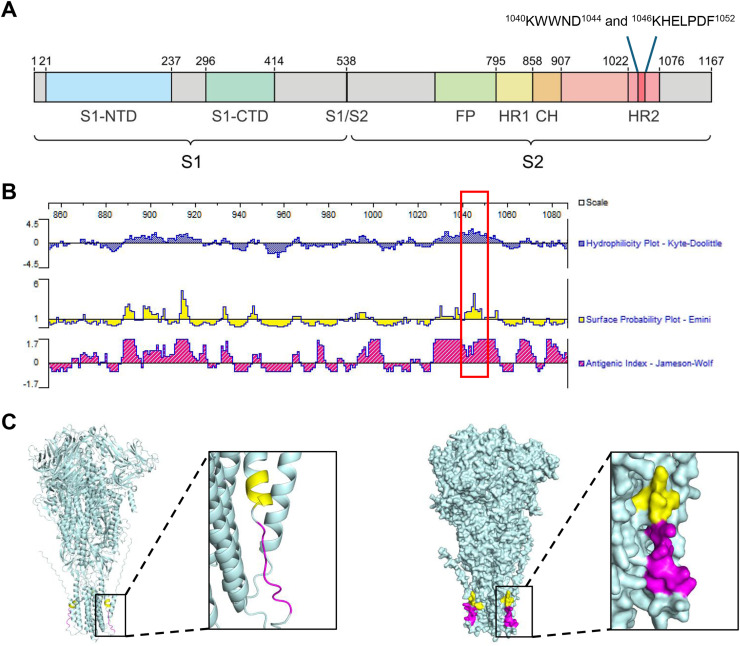
Bioinformatics analysis of epitopes. (A) Schematic diagram of IBV spike protein domains. S1-NTD: N-terminal domain of S1 subunit; S1-CTD: C-terminal domain of S1 subunit; S1/S2: cleavage site between S1 and S2 subunits; CH: central helix; FP: fusion peptide; HR1: heptad repeat 1; HR2: heptad repeat 2. (B) Analysis of antigenicity, hydrophobicity, and surface probability of S_860–1088_ using the Protean software. (C) Mapping of the epitopes on the three-dimensional structure of the full-length spike protein. The epitope ^1040^KWWND^1044^ is shown in yellow, while epitope ^1046^KHELPDF^1052^ is shown in purple.

The epitopes recognized by the mAbs were located within the HR2 of the spike protein S2 subunit (Fig. 4A). The highlighted epitope regions showed high hydrophilicity, high surface accessibility, and strong antigenicity (Fig. 4B). The three-dimensional structure of the spike protein was predicted using AlphaFold3 and visualized using PyMOL software. Structural analysis demonstrated that the ^1040^KWWND^1044^ epitope was located within an α-helical region, while the ^1046^KHELPDF^1052^ epitope was situated within a random coil domain. Notably, the three-dimensional structure of the protein also showed that both epitopes are exposed on the protein surface (Fig. 4C).

### 3.5 Cross-reactivity assessment of mAbs across different viral strains

**Fig. 5:**
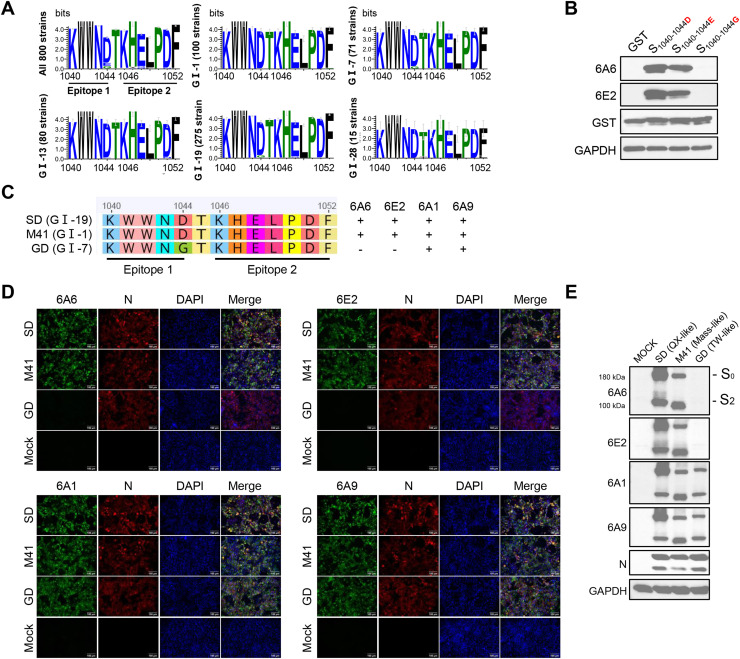
Evaluation of epitopes conservation and mAbs cross-reactivity among different IBV strains. (A) Amino acid sequences of the corresponding epitope from different lineages of IBV were aligned using Geneious Prime software, and sequence conservation logos were generated using WebLogo. The y-axis shows information content (in bits), indicating the degree of conservation at each position. (B) Effect of amino acid variation at residue 1044 on monoclonal antibody recognition. Vero E6 cells were harvested 24 h after transfection, and the reactivity of mAbs 6A6 and 6E2 against different amino acid variants was analyzed by WB, with GAPDH as an internal control. (C) Epitope sequences of three representative IBV strains. "+" indicates a positive reaction; "-" indicates a negative reaction. (D) IFA showing the reactivity of mAbs against different IBV strains. Cells infected at an MOI of 0.1 were fixed at 36 hpi and stained with antibodies against spike (green) and nucleocapsid (N, red) proteins. Scale bar: 100 μm. (E) WB analysis of mAbs reactivity against different IBV strains. Cells infected at an MOI of 0.1 were harvested and lysed at 24 hpi. mAbs reactivity against different strains was analyzed by WB. GAPDH served as an internal control, and N protein as a loading control.

Several lineages mentioned above were selected from 800 IBV strains for comparative analysis of the spike protein amino acid sequence at positions 1040–1044 and 1046–1052. This region was highly conserved across different IBV lineages, with amino acid variation detected only at position 1044, where three variants were identified: aspartic acid (D), glutamic acid (E), and glycine (G) (Fig. 5A). To determine how amino acid variation at position 1044 affects monoclonal antibody recognition, we constructed variants of the antigenic epitope ^1040^KWWND^1044^ by replacing the D at position 1044 with E and G. Variants were cloned into pCMV-FLAG-GST vector and expressed in Vero E6 cells. WB showed mAbs 6A6 and 6E2 recognized the 1044D and 1044E variants but not the 1044 G variant (Fig. 5B). Despite loss of binding to 1044 G variants, 6A6 and 6E2 still recognize 91.3 % of strains in our dataset, showing good broad-spectrum reactivity. To further validate these results, three representative IBV strains—SD (GI-19), M41 (GI-1), and GD (GI-7)—were selected for comparison, along with their corresponding key antigenic epitope sequences (Fig. 5C). IFA results showed the cross-reactivity of different mAbs against various IBV strains. MAbs 6A6 and 6E2 reacted specifically with SD and M41 but failed to recognize the GD strain, which was attributed to the presence of G at position 1044 in the GD strain, in agreement with the plasmid-based validation results (Fig. 5D). In contrast, mAbs 6A1 and 6A9 exhibited broader cross-reactivity, showing positive reactions with all three representative strains, indicating that these antibodies recognize more conserved antigenic epitopes. WB analysis further confirmed the IFA results, showing consistent recognition patterns across both assays (Fig. 5E). Overall, these findings indicate that, despite the amino acid variation at position 1044, the two epitopes remain highly conserved among different IBV lineages, allowing the generated mAbs to retain broad cross-reactivity against diverse strains.

## 4 Discussion

Over the past 90 years, IBV has become a major threat to the global poultry industry, causing hundreds of millions of dollars in economic losses annually by reducing growth performance, decreasing egg production and quality, and increasing mortality rates in chickens (Abozeid, 2023; Cook et al., 2012). Therefore, developing effective diagnostic methods for serological surveillance of IBV is essential for disease control. As the major surface glycoprotein, the spike protein plays a central role in viral infection. Its surface-exposed structure and strong immunogenicity make it an ideal target for serological detection (Legnardi et al., 2020; Xie et al., 2023).

Unlike previous studies that focused mainly on epitopes within the S1 subunit of the spike protein, our research examined the more conserved S2 subunit (Gao et al., 2025; Sives et al., 2023; Zou et al., 2015). In this study, we selected a conserved region within the IBV S2 subunit (860–1088 aa) for prokaryotic expression and purification. We immunized mice with the purified recombinant protein and successfully obtained four monoclonal antibodies (6A6, 6E2, 6A1, and 6A9) that specifically recognize the S2 protein. By constructing a series of truncated expression fragments, we mapped the epitopes recognized by these mAbs to two adjacent, previously unreported linear epitopes: ^1040^KWWND^1044^ and ^1046^KHELPDF^1052^. Bioinformatics analysis suggested that the two epitopes are exposed on the spike surface and have good immunogenicity, making them likely to elicit antibody responses.

Both epitopes are located within the HR2 region of the S2 subunit. During viral entry, this region forms a six-helix bundle (6-HB) structure with HR1, which is critical for viral membrane fusion (Li et al., 2025). We further performed neutralization assays, but none of these four mAbs showed neutralizing activity (data not shown). We propose that these mAbs target linear epitopes in the HR2 region rather than conformational epitopes, and thus lack the ability to stabilize the prefusion state and prevent membrane fusion. This finding is consistent with previous reports that mAbs against linear epitopes in the HR2 region of SARS-CoV-2 also show low and incomplete neutralizing efficacy (Planchais et al., 2024). In contrast, antibodies targeting quaternary epitopes in HR2 demonstrate potent neutralizing activity by binding two adjacent HR2 α-helices, locking the prefusion conformation, and preventing HR2 migration toward HR1 and 6-HB formation (De Cae et al., 2025). The recognition of only linear epitopes by our antibodies may be due to the use of inclusion body protein as the immunogen. Inclusion body proteins are typically denatured or misfolded and lack the three-dimensional structure of native proteins. Their linear peptide segments are fully exposed and become the primary targets for immune recognition (Baneyx and Mujacic, 2004; Ratanji et al., 2014). This suggests that future studies should use natively folded proteins to generate antibodies with greater neutralizing potential against viral entry at early infection stages. Although these mAbs lack neutralizing activity, their specific recognition of HR2 linear epitopes can be used as immunological probes to map the spatial distribution of S2 subunit during infection and its potential interactions with host factors, contributing to a deeper understanding of S2′s role in viral infection process.

Beyond these research applications, binding antibodies are also valuable for diagnostic assay development, and their utility is determined by the breadth of epitope recognition, which motivated us to assess epitope conservation. Sequence alignment showed that both epitope sequences are highly conserved among different IBV strains, with variation mainly limited to position 1044 (D, E, or G). Although mAbs 6A6 and 6E2 cannot recognize the 1044 G substitution, they still cover 91.3 % of strains. In contrast, mAbs 6A1 and 6A9 show broader cross-reactivity. The S1 subunit has traditionally been the focus of vaccine development due to its abundance of neutralizing epitopes (Zou et al., 2015). However, its high mutation rate limits its use in serological diagnostics. These conserved linear epitopes provide a foundation for developing broad-spectrum serological diagnostic methods. Peptide-based ELISAs can be constructed directly using the conserved peptide segments (Yin et al., 2021). Alternatively, mAbs prepared against these conserved epitopes can serve as specific capture or detection antibodies in ELISA methods, enabling broad-spectrum recognition of different IBV (Liu et al., 2019). In our previous study, we developed a quantum dot-based immunochromatographic assay using a mAb against the SABD domain of the S1 subunit as one of the capture antibodies (He et al., 2025). Replacing it with monoclonal antibody 6A1 or 6A9 could further enhance the broad-spectrum capability of this detection method.

In conclusion, this study successfully generated four specific mAbs and identified two novel, conserved linear B-cell epitopes (^1040^KWWND^1044^ and ^1046^KHELPDF^1052^) in the HR2 region of the IBV spike protein S2 subunit. These findings will help further study the biological properties of IBV spike protein and provide valuable resources for developing broad-spectrum IBV diagnostic reagents.

## CRediT authorship contribution statement

**Liwei Zhang:** Writing – original draft, Software, Methodology, Formal analysis, Conceptualization. **Yingfei Li:** Writing – original draft, Visualization, Software, Methodology, Formal analysis, Conceptualization. **Xuehui Zhang:** Visualization, Software, Methodology, Formal analysis. **Jing Zhao:** Validation, Project administration, Formal analysis. **Guozhong Zhang:** Writing – review & editing, Supervision, Resources, Project administration, Conceptualization. **Ye Zhao:** Visualization, Supervision, Project administration, Formal analysis, Conceptualization.

## Declaration of competing interest

The authors declare no competing interests.

## Data Availability

Data will be made available on request.
